# Supplementary material for: Quantifying the COVID19 infection risk due to droplet/aerosol inhalation
Source: Sci Rep. 2022 Jul 1;12:11186. doi: 10.1038/s41598-022-14862-y (PMC9249924; doi:10.1038/s41598-022-14862-y)
Supplement: Supplementary file 1 — Supplementary Information. [file 41598_2022_14862_MOESM1_ESM.pdf]

Supplementary Information: Quantifying the COVID19 infection risk due to droplet/aerosol inhalation

Rahul Bale[1,2,*], Akiyoshi Iida[3], Masashi Yamakawa[4], ChungGang Li[1,2], and Makoto Tsubokura[1,2]

[1]Riken Center for Computational Sciences, Japan
[2]Graduate School of System Informatics, Kobe University, Kobe, Japan
[3]Department of Mechanical Engineering, Toyohashi Institute of Technology, Toyohashi, Japan
[4]Department of Mechanical Engineering, Kyoto Institute of Technology, Kyoto, Japan
[*]Corresponding author: E-mail: rahul.bale@riken.jp

Grid Convergence

The numerical simulation of the speaking flow was carried out with three different meshes in which the mesh spacing in the a region of at least $1m \times 0.5m \times 0.5m$ along the axial, lateral and vertical directions, respectively, in front of the mouth geometry from which the speaking flow emanates. The mesh spacing of the three meshes in this region were 8mm (coarse), 4mm (medium) and 2mm (fine), respectively. As the distance from the mouth increases the mesh spacing progresively decreases for any given mesh. For example, for the medium mesh, the mesh spacing increase from 4mm to 8mm, then from 8mm to 16mm and so on as the cube sizes are changed (See Fig. 3 of the manuscript). The time averaged axial velocity along the center line of the speaking jet flow is plotted for the three meshes in Fig. 1a. The simulations were carried out for a physical time of 80s for three cases and the time averaging of the data was done for the same period. We find that there is no significant variation between the three results. Therefore we proceed adopt the fine mesh for the present study. In Fig. 1b, a comparison of the scalar concentration is presented for the three meshes. The scalar concentration is derived from the mass fraction of the vapor phase of the liquid droplets(Y_d), which is defined as follows

$$\overline{Y}_s = \frac{\overline{Y}_d - Y_d^{\infty}}{Y_d^{o} - Y_d^{\infty}} \tag{1}$$

where the overbar indicates a time-averaged quantity, Y_d^{∞} and Y_d^{o} are the ambient mass fraction of the vapor phase of the droplet and that at the source of the injection.

Solver framework and simulation environment

A multi-physics solver known as CUBE[1, 2] has been used for all the numerical simulations presented in this work. CUBE is a finite volume solver based on a hierarchical meshing framework known as the building cube

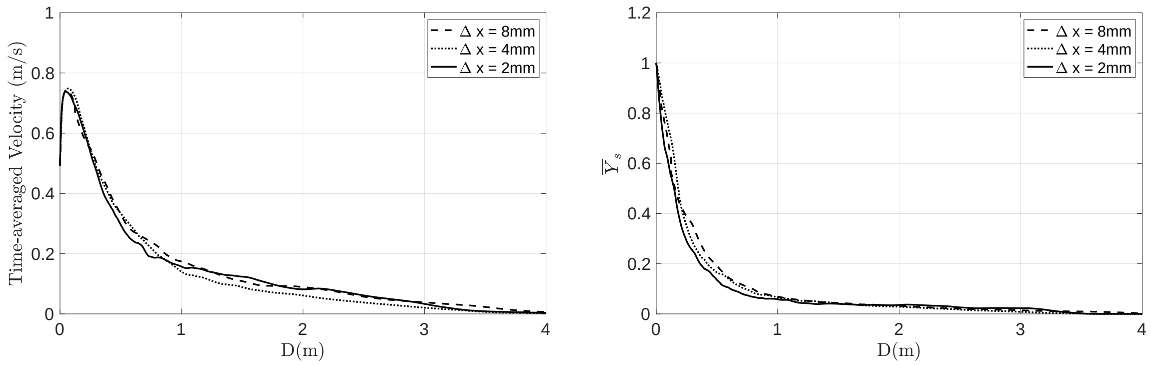

(a)

(b)

Figure 1: (a) Comparison of axial velocity along the axial center line of the speaking jet flow for the coarse, medium and fine meshes. (a) Same as (a) with the comparison of the scalar concentration.

method (BCM)[3]. The meshing framework allows local mesh refinement enabling the high resolution in regions of interest while limiting the overall cell count. The supercomputer Fugaku has been used for carrying out the numerical simulation presented in this work. Fugaku comprises 158,976 nodes. Each node is equipped with a Fujitsu A64FX processor, which consists of 48 compute cores and 4 additional cores, and a memory of 32 GiB. The nodes are interconnected with 28Gbps, 2 lanes, 10 port TofuD interconnect.

Infection probability post vaccination

The probability of infection can be written as

$$P = 1 - e^{(-\frac{N(x,t)}{N_0})} = 1 - \exp^{(-\frac{N_c}{N_0}t)} \tag{2}$$

where N_c is the virion dose per unit time under steady state conditions of exposure such as speaking, singing etc. The exact form of N_c is not pertinent to the present derivation, it can deduced from Eq. **??**. The probability of no infection can be defined as $\hat{P} = 1 - P$. The net probability of no infection is given by

$$\hat{P}_{net} = \int_0^{\infty} \exp^{(-\frac{N_c}{N_0}t)}\, dt$$
$$\hat{P}_{net} = \frac{N_0}{N_c} \tag{3}$$

Assuming that the vaccination leads to reduction in infection probability due to increase in N_0, the net probability of no infection for a vaccinated individual can be expressed as

$$\hat{P}^{vc}_{net} = \frac{N^{vc}_0}{N_c} \tag{4}$$

Let η_{vc} be the efficacy of a given vaccine. The probability of no infection with vaccination decreases by a factor of $\hat{P}^{vc}_{net}\eta_{vc}$ if the vaccination is not administered. Therefore, we can express a relationship between $\hat{P}_{net}$ and $\hat{P}^{vc}_{net}$ as follows

$$\hat{P}_{net} = \hat{P}^{vc}_{net} - \hat{P}^{vc}_{net}\eta_{vc}. \tag{5}$$

Rearranging the terms we get

$$\hat{P}^{vc}_{net} = \frac{\hat{P}_{net}}{1-\eta_{vc}}. \tag{6}$$

Assuming an exposure to the same average virion dose per unit time, we can obtain an expression for N_0^{vc} from the above two equation as

$$N_0^{vc} = \frac{N_0}{1-\eta_{vc}} \tag{7}$$

The interpretation of the above two equations is straightforward. If the vaccine efficacy the 100% then the minimum number of virions needed for infection will be ∞. Consequently, $\hat{P} = 1$ at all time instants t and $P = 0$ and the net probability of no infection will be ∞. On the other hand, if the vaccine efficacy is 0 then the $N_0^{vc} = N_0$. Therefore, probability of infection remains unchanged.

The general form of the probability of infection under vaccinated and unvaccinated situations can be expressed as

$$P = 1 - e^{(-\alpha \frac{N}{N_0})}, \tag{8}$$

where $\alpha = 1$ for no vaccination cases and $\alpha = 1 - \eta_{vc}$ for vaccinated cases.

The net probability of safety decreases for the variants strains due to higher transmissibility τ compared to the original strain. With $\hat{P}^{vr}_{net}$ representing the probability of no infection, its relationship with $\hat{P}_{net}$ can be written as

$$\hat{P}^{vr}_{net} = \frac{\hat{P}_{net}}{\tau} \tag{9}$$

Along the same lines of derivation of α for the vaccination case, it can be shown that $\alpha = \tau$.

Bibliography

References

[1] N. Jansson, R. Bale, K. Onishi, M. Tsubokura, Cube: A scalable framework for large-scale industrial simulations, The International Journal of High Performance Computing Applications 1094342018816377.

[2] K. Nishiguchi, R. Bale, S. Okazawa, M. Tsubokura, Full eulerian deformable solid-fluid interaction scheme based on building-cube method for large-scale parallel computing, International Journal for Numerical Methods in Engineering 117 (2) (2019) 221–248.

[3] K. Nakahashi, Building-Cube Method for Flow Problems with Broadband Characteristic Length, in: Computational Fluid Dynamics 2002, Springer Berlin Heidelberg, 2003, pp. 77–81.
